# Supplementary material for: Dorsal and median raphe neuronal firing dynamics characterized by nonlinear measures
Source: PLoS Comput Biol. 2024 May 28;20(5):e1012111. doi: 10.1371/journal.pcbi.1012111 (PMC11161118; doi:10.1371/journal.pcbi.1012111)
Supplement: Attachment — Submitted filename: ResponsesToReviewers.pdf [file pcbi.1012111.s003.pdf]

Montevideo, February 21st, 2024

Dr. Claudio R. Mirasso
Guest Editor
PLOS Computational Biology

Dr. Thomas Serre
Section Editor
PLOS Computational Biology

Dear Dr. Mirasso and Dr. Serre,

We thank both referees, and the Editors, for the time taken to critically review our manuscript, No. PCOMPBIOL-D-23-01259. We are enclosing the revised version of it, entitled “Dorsal and median raphe neuronal firing dynamics characterized by nonlinear measures”, and our point-by-point reply.

We have addressed all the comments and issues raised by the reviewers, modifying the manuscript accordingly. We think that the comments and suggestions have made the paper stronger and we are looking forward to a positive decision in the near future. We thank the reviewers again for their time taken in reading and commenting on our manuscript and engaging in scientific interaction.

Yours faithfully,

The authors

Claudia Pascovich, Diego Serantes, Alejo Rodriguez, Diego Mateos, Joaquín González, Diego Gallo, Mayda Rivas, Andrea Devera, Patricia Lagos, Nicolás Rubido, and Pablo Torterolo.

Av. Gral. Flores 2125 - 11800 Montevideo Uruguay
- (598) 29243414-3234

REVIEWER 1

[…]. The quality of the analysis presented in this work is outstanding, and I do believe in the added value of non-linear metrics.

Response A: We are grateful to the Reviewer for his/her assessment and the time taken to carefully review our manuscript.

[…]. The type of neurons compared is unknown. They belong to two different nuclei (DRN and MRN), but they contain a rich variety of neuron subtypes (Huang et al., 2019). Thus, the results cannot be directly associated to the type of neurons but to their location (DRN or MRN). My question is: why is it useful to differentiate between DRN and MRN neurons? A good experimental procedure, as the one presented in this work, would separate them.

Response B: First and foremost, it is essential to highlight that the primary objective of the manuscript is to assess novel non-linear metrics for distinguishing neuronal populations. We aim to compare these metrics with classical tools traditionally used for characterizing neuronal firing patterns. To achieve this goal, we analyzed neurons from both the DRN and MRN, two neuronal groups that have undergone thorough examination within our research group.

Secondly, the Reviewer is correct: neurons in our study come from different nuclei but their type is unknown, and there is a wide variety of subtypes in each nuclei (DRN and MRN). We lack the necessary information from our experiments and dataset to classify different neurochemical phenotypes, such as serotonergic and GABAergic neurons. We opted to test nonlinear measures in neurons belonging to two anatomically and functionally different nuclei of the raphe, which have been extensively studied with classic/traditional linear measures. Hence, our results can be associated with the location of the neurons, which we tried to highlight across the revised version of the manuscript.

Regarding the question, it is useful to characterize the dynamics of neurons from the DRN (dorsal raphe nucleus) and MRN (median raphe nucleus) because these two nuclei (located in the brainstem) play distinct roles in neurotransmission and physiological functions. For example, the microinfusion of a selective agonist of the 5-HT1A receptor (8-OH DPAT) into the MRN, which inhibits the activity of serotonergic neurons, produces general behavioral hyperactivity, contrary to what occurs in the DRN with a decrease in certain behaviors, such as rearing and grooming (Salles and Salles, 1980). Regarding pain control, electrolytic lesions of the MRN antagonize morphine analgesia, whereas destruction of the serotonergic neurons in this nucleus does not produce such effect, indicating that the substrate mediating this action of the nucleus remains unknown (Romandini et al., 1986). However, lesions of the DRN increase reactivity and aggression evoked by pain, while lesions of the MRN do not have such effect (Jacobs and Cohen, 1976; Przewlocka et al., 1977).

It is important to highlight that the function of each nucleus depends on the action of multiple neurotransmitters. Moreover, both nuclei can contain the same neurotransmitters but in different proportions, which does not make them functionally equivalent. The function of each neuron also depends on a multitude of other local factors, including its

connections: the DRN and MRN have different projections, and in turn, receive afferents from different regions.

A comparison of the projections of the DRN and MRN shows that these two large groups of neurons are distributed in regions that, in most cases, do not overlap in the anterior brain; instead, they project to complementary regions. For example, the DRN projects to the medial prefrontal cortex, the amygdala, and the accumbens nucleus, whereas the MRN projects to medial structures, including the medial septum and diagonal band, zona "incerta", and posterior hypothalamus (Vertes et al., 1999, Commons et al., 2016).

In summary, the differentiation between DRN and MRN neurons is crucial for a more nuanced understanding of their roles in neurobiology, behavior, and various physiological functions.

We have modified the Introduction to highlight these points, and shifted the discussion on the serotonin importance.

As the proportion of serotonergic neurons is much higher in the DRN, the way the manuscript is written suggests that non-linear metrics may identify these neurons, but this cannot be inferred from the analysis and it is not explicitly said (except in section 2.5, but it is another topic). The text revolves around the importance of serotonergic neurons, but most of the results are not linked to them, which is confusing. The relation between non-linear metrics and serotonergic neurons beyond section 2.5 should be clearly stated.

Response C: Our work focuses on finding distinct characteristics between DRN and MRN neurons from linear and nonlinear measures (as we discuss in Response B), analyzing the redundancy of these measures to characterize neuronal dynamics (i.e., if the measures are functionally related or if they provide complimentary information), finding which measures have values that differ between clock-like and non-clock-like rhythmic neurons, and assessing the sensitivity of the measures to the presence of burst patterns in the inter-spike interval dynamics. We thank the reviewer for raising these issues, since it has helped us to clarify our work, modifying the manuscript accordingly.

Specific concerns

1. Goal and limitations of the analysis. As mentioned before, it is not clear the utility of differentiating neurons from two spatially separated nuclei. If the goal is to differentiate serotonergic vs. non-serotonergic neurons, this should be clearly stated in the text. As I think the authors cannot directly relate their results with serotonergic neurons, they should better describe and discuss why they may be related and the limitations of their approach.

R1: Our goal is to test if the nonlinear measures are useful to differentiate the neuronal dynamics of two functionally different groups of neurons, regardless of their specific neurochemical phenotype and in contrast to the classical/typical linear measures. We have clarified this in the revised manuscript, as well as the importance of differentiating between DRN and MRN neurons (see Response B). We also shifted the focus away from

Av. Gral. Flores 2125 - 11800 Montevideo Uruguay
- (598) 29243414-3234

serotonergic neurons in the Introduction, and tried to improve the discussion of our results on serotonergic versus non-serotonergic neurons, highlighting the limitations in our methodology.

2. The proportion of the main types of neurons in DRN and MRN from previous work should be included and discuss which may be the neurons driving the results.

R2: As the Referee correctly mentioned, our work is not focused around the classification of serotonergic and non-serotonergic neurons, but in the characterisation of neurons from the DRN and MRN. Consequently, we have revised the manuscript to highlight the importance of this characterisation and moved to the Discussion the serotonergic neurons, their importance, and how these neurons could be driving some results.

We are confident that the differences in OP Entropy from the DRN and MRN are not due to clock-like putative serotonergic neurons, because our analysis shows that these neurons have similar OP entropy values as those from the rest of the population. However, this comparison has the limitation that the method we used for selecting the clock-like putative serotonergic neurons is not based in immunochemistry but on the classic linear measures, therefore our results in this matter could be contested.

3. Section 2.1. How the metrics are related is not explained in the methods section. Is it linear correlation? Information about the stats is missing (r2, degrees of freedom…). Which are the best features to differentiate the neurons. Is the inclusion of a second parameter really adding information?

R3: We agree with the Reviewer that our methodology needed more explanation and details, particularly regarding the scatter plot (Fig. 3), model fitting (Fig. 4), and statistical tests (Table 1), which we have now modified accordingly in the revised version of the manuscript.

The results in Fig. 3 show a scatter plot of the values from the measures for DRN and MRN neurons, which are highlighted by shaded areas. We have clarified in the revised manuscript that we are not doing a classification or clustering analysis here. We are visually describing the paired values of the linear and nonlinear measures to show qualitatively how some pairs of measures have values that are different between the nuclei and some pairs have no differences between the nuclei.

The statistics in Table 1 come from applying linear mixed effect (LME) models between each pair of measures (Fig. 4), which is done to take into account the multilevel structure of the dataset. The LME models are a type of linear regression with nested structure, in which some of the predictors are not independent. In our case, the neurons are clustered in rats because a certain group of neurons had been recorded in one rat, while another group of neurons were recorded in a second rat, so these recordings create a nesting structure that introduces a variance that has to be taken into account. This is now specified in the revised Methods.

The fitting in Fig. 4 and results from Table 1 (see new table below) correspond to linear and nonlinear model fittings (not correlation) and the Bayes Factor as the statistical test (which due to changes suggested by

reviewer 2, we have now removed the Bayes Factor). Our goal here is to assess whether we can find a functional relationship between 2 measures (be it linear or nonlinear), which would imply the existence of redundancy between the measures. This means that if we cannot find a linear or nonlinear fitting between 2 measures, then we can conclude that these 2 measures provide complementary/independent information.

We have now changed Fig. 4 to include a linear or nonlinear fitting only when it exists (as suggested by the reviewers). To do this, we looked for a linear fitting on each paired measure when plotted in a linear scale, and looked for another linear fitting on the paired measures when plotted in a log-log scale, which corresponds to fitting a (nonlinear) power-law. We keep whichever fitting is best, and discard those that are not significant. As suggested by the reviewer, we now have also calculated the Pearson correlation coefficient r for the pairs of measures which we found an optimal fitting (either in the linear or logarithmic scale).

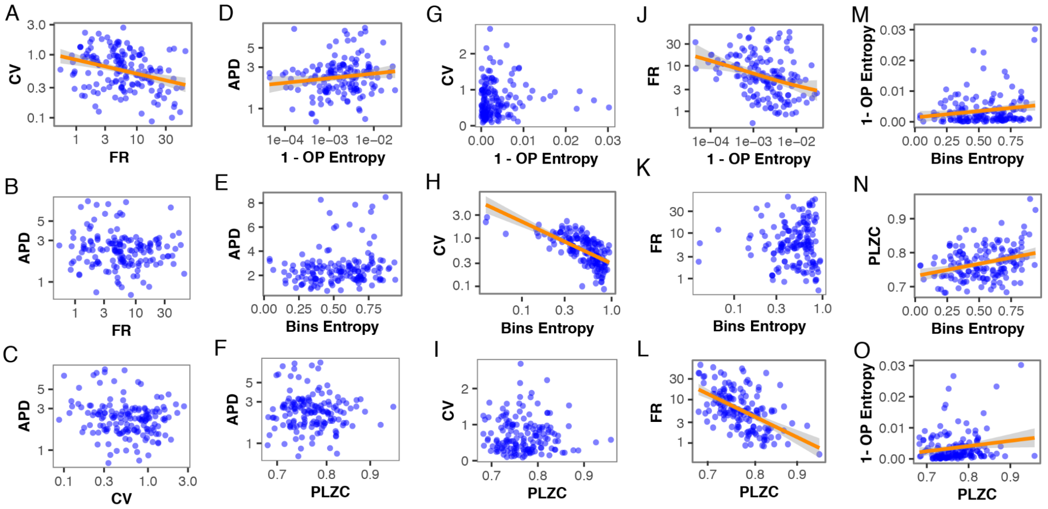

(New) **Figure 4** – The best fit for the values in panel A happens in log-log scale, meaning that a nonlinear relationship exists between CV and FR, with an exponent β_A = -0.50 (i.e., CV proportional to FR^{β}) and a Pearson correlation coefficient of r = -0.32. Similarly, the best fit for the values in panels D, H, J, and L also happens in the log-log scale, where the respective exponents are β_D = 0.46 [i.e., APD ∝ (1 - OP entropy)$^{\beta}$] with r = 0.18, β_H = -1.59 [i.e., CV ∝ (bin entropy)$^{\beta}$] with r = -0.65, β_J = -53.55 [i.e., FR ∝ (1 - OP entropy)$^{\beta}$] with r = -0.25, and β_L = -9.27 [i.e., FR ∝ (PLZC)$^{\beta}$] with r = -0.54. Contrary, the best fit for panels M, N, and O happens in a linear scale, meaning linear relationships of slopes α_M = 4.11x10-3 with r = 0.17 , α_N = 0.07 with r = 0.31, and α_O = 0.02 with r = 0.16, respectively.

	Mean Freq.	CV	APD	OP Entropy	Bins Entropy	PLZC
Mean Freq.		< 0.001***	0.338	0.001**	0.864	< 0.001***
CV	< 0.001***		0.136	0.132	< 0.001***	0.909
APD	0.338	0.136		0.0192*	0.169	0.427
OP Entropy	0.001**	0.132	0.0192*		0.024*	0.035*
Bins Entropy	0.864	< 0.001***	0.169	0.024*		< 0.001***
PLZC	< 0.001***	0.909	0.427	0.035*	< 0.001***	

(New) **Table 1** – Statistical comparison between measures using linear multilevel models. F statistic t-test is used to test the linear relationship between the fixed effects. *p < .05, **p< .005, ***p <.001.

Regarding which is the best feature to differentiate the neuronal populations, our results from Fig. 2 show that the OP Entropy is the only one that can differentiate DRN and MRN neuronal populations. When classifying the neuronal populations according to rhythmicity, then the APD and bin entropy are the best features to statistically differentiate rhythmic neurons from the DRN and MRN (Fig. 5). However, neither of these features can be assigned to a single-neuron as a specific measurement that can classify the neuron into a particular nucleus. The reason is that we work with distributions (i.e., populations) and their differences, and not with a diagnostic/clinical tool for a single neuron.

Regarding the inclusion of a second parameter and the addition of information, we note the following. In other works, various features have been used (such as the CV, APD, and average spiking frequency), but an analysis of redundancy was missing. Namely, assessing how much information is repeated from one feature to the other. Figure 4 (old and new) intends to show which of the linear and nonlinear features provide redundant information and which provide complementary/independent information (regardless of the nuclei). For example, we find a significant (Table 1) nonlinear relationship between the CV and mean frequency FR (Fig. 4A) as well as between the CV and Bins Entropy (Fig. 4H). This means that the dynamical characteristics revealed by the FR or Bins Entropy values are contained in the CV values, rendering their joint use irrelevant/redundant. However, we could not find a linear or nonlinear relationship between the CV and OP entropy (Fig. 4G), making these 2 measures complementary (i.e., they add information).

We have reviewed the manuscript to clarify these points, including the missing information of the methods and the reviewer's suggestions.

4. What is the percentage of neurons correctly classified as DRN or MRN in the best-case scenario?

R4: Our aim is to improve the characterization of the neurons from the DRN and MRN at a population level. So, our approach does not allow us to define a single-neuron classification method, which would allow us to do a ROC (Receiver Operating Characteristic) analysis and get a "best-case scenario" for each tested neuron. Instead, our approach is to take both populations of neurons as a whole (distribution), and find the main statistical characteristics that differentiate their dynamical properties at a population level.

5. Figure 4. Difficult to say which are significant from the figure. Maybe include the linear regression only in those that are significant.

R5: We thank the reviewer for this suggestion, which we have modified Fig. 4 accordingly (and as suggested by reviewer 2). Please see **R3**.

6. There are serious limitations in section 2.5. Putative neurons are selected based on linear features and only 7 out of 66 are labeled as serotonergic. (i) Where is the 66 coming from? (ii) The proportion is quite low, suggesting that many serotonergic neurons were incorrectly labeled and non-serotonergic. (iii) The populations are defined by linear metrics, so the differences between them are linear. Indeed, the main non-linear metric separating both groups is the Bins Entropy, which highly correlates with the linear features. (iv) Only two sentences in the discussion about this analysis (293-295), just to mention the result. What is the goal of this section?

R6: (i) 66 is the total number of neurons recorded in the DRN.
(ii) We are aware that other serotonergic neurons may be incorrectly labeled as non-serotonergic because other subgroups with more varied characteristics have been described in the DRN. However, we don't have a way to distinguish this subgroup of (possibly serotonergic) neurons without immunohistochemistry. Our way of labeling the neurons as putative serotonergic is based on considering APD larger than 1.4, CV lower than 0.30, rhythmic pattern in the ACH, and low FR (<4Hz). This implies that we can only label neurons as putative serotonergic if they have a classic "clock-like" spiking. We have now stressed this in the revised manuscript.
(iii) We agree that because we are using linear metrics to separate both groups (i.e., clock-like serotonergic from non-clock-like serotonergic), the differences between groups are expected to be linear. However, nonlinear metrics should be able to find linear differences as well as nonlinear differences. Hence, our results from Fig. 7 are not trivial – we were expecting to find differences when using OP entropy and PLZC as well. In particular, we were expecting to have low entropy values for the clock-like serotonergic neurons. This needs further investigation, which is outside the scope of our current work.
(iv) The goal of Section 2.5 is to add information to the characterisation of the spiking dynamics from neurons that are clock-like and those that aren't. Aside from the linear characteristics provided by the AP, FR, and CV, we test in this Section which are the entropic characteristics of the spiking sequences that could differentiate the dynamics between clock-like and non-clock like activity. We have clarified this in the revised version of Section 2.5 and extended the Discussion about this topic to add this information.

7. The Bayes Factor is not explained in the methodology. More information and citations in section 4.4 would be appreciated.

R7: Given the changes done to Fig. 4 (according to the suggestions by both reviewers), we removed the Bayes Factor from our analysis. In general, the Bayes Factor can be used to compare model fitting and decide if there is more evidence in favor of one or the other model fitting.

Av. Gral. Flores 2125 - 11800 Montevideo Uruguay
- (598) 29243414-3234

Information can be found in (Makowski, D., et al. (2019)). In the previous version, we had used the package (bayestestR) in RStudio and contrasted two models. A Bayes factor greater than 1 can be interpreted as evidence against the null model, where the convention is that a Bayes factor greater than 3 can be considered as "substantial" evidence against the null model, and vice versa, a Bayes factor smaller than 1/3 indicates substantial evidence in favor of the null-model (Wetzels et al. 2011).

Minor Comments

R: We thank the referee for spotting the following mistakes, which we have corrected in the revised manuscript.

Line 94: APD of 3.07 +- 0.37ms. Is it mean +- standard error of the mean?
R1m: Yes it is**.**

Line 95: ACH and IH should be defined here.
R2m: We have defined them now when they first appear.

Line 110: Figure 2A (There is only one panel).
R3m: Corrected that in the revised manuscript.

Line 294: Figure 6B (It is 7B).
R4m: Corrected that in the revised manuscript.

Line 373: Why Nb=18 has the same statistical power as the OP entropy?
R5m: The reason stems from the use of D = 3 for the OP encoding, which we chose because of the shortness of the Inter-Spike Interval (ISI) series. When using D = 3, we can have 6 different OP symbols (different permutations). This means that, a sequence of N ISIs, would give us a sequence of (approximately) N/3 OPs, which would then be divided into 6 bins corresponding to the different OP symbols. On average, that would give us N/3/6 = N/18 symbols per bin. On the other hand, for the bin entropy we are able to use the complete ISI series of N values. This means that a histogram of the ISI values with 18 bins would in average contain the same number of ISI values per bin as the histogram of OP symbols. This clarification is now in the revised Methods, Section 4.3.

Was the multi-unit activity (MUA) excluded from the analysis?
R6m: In this study we recorded the neurons by single neuron recording – not by a multi-unit recording.

What is the duration of the recordings?
R7m: The duration of the recordings varies between 1 to 5 minutes sampled at 11kHz. However, as we used the ISI series, the number of datapoints depends on the frequency of the neuronal spiking activity. In neurons with a high spiking frequency we had hundreds of ISI values in a minute, whereas we needed more time to have the same number of ISIs for a neuron discharging in a low frequency. To make our analysis uniform, we fixed the spiking signals for all neurons to 225 ISI values, cutting short the longer ISI sequences.

How many rats received Nb?

R8m: 14 rats. This information is now included in the revised version of the manuscript.

REVIEWER 2

1. The ISI time series can be understood as a mapping of the dynamic. That justifies the use of a lag = 1 (consecutive ISI). Yet, larger order correlations or characteristics of temporal scales can be found when larger lags are used. For instance, I wonder, does the permutation entropy go to 1 when the lag increases? When analyzing the walk of the ISI sequence, is there self similarity?

Response 1: Indeed, we are only considering a lag = 1 mapping, and it is important to consider higher lags to account for higher-order correlations. However, we have data restrictions that limit our analysis: our single-neuron Inter-Spike Interval (ISI) sequences contain only 225 ISI values (see our reply R7m to reviewer 1). This means that when doing the OP encoding with D = 3, we have 225/3 = 75 non-overlapping embedded windows that are transformed to an OP. Since with D = 3 we can have D! = 6 possible OP symbols, then each OP can appear on average 75/6 = 12.5 times. In order to improve this value, we encoded the ISI sequence twice, first starting in the initial ISI value, and then starting in the second ISI value. Using both of these encoded sequences, we calculated the probability distribution of OPs, which in average has 2x75/6 = 25 symbols per possible OP symbol. Because of this limitation, we can neither extend our OP analysis to D = 4 nor do higher lags.

Even if we would consider overlapping embedding, we would end with ~225 OPs with D = 3, which would mean an average number of symbols per OP of 225/6 = 37.5. This would improve our confidence in the OP statistics slightly, but it would not be enough to carry an embedding delay analysis (i.e., increasing the lag to have sufficient values of the permutation entropy as a function of the lag for an extrapolation that would give us the entropy rate of the system).

We have now included in the manuscript the missing information about the number of ISIs, the double OP encoding in the revised Methods Section 4.3, and talked about the choices we made and limitations of our OP analysis in the revised Discussion, Section 3.5.

2. I think that a discussion of which dynamical features are found or at least expected to be found is missing. The values of the permutation entropy are pretty high, then, the observed dynamic is a stochastic noise? Is it temporally correlated? Are there nonlinear features?

R2: We have added a discussion of the dynamical features, in relation to past works and our expectations.

We agree that the permutation entropy is high (which was also puzzling for us, particularly for the clock-like neurons), but we checked that the ISI sequences resemble a stochastic process (even for the clock-like neurons). The reason is that when differentiating neurons between rhythmic and non-rhythmic, we use the classical approach of analyzing the event autocorrelation (i.e., the aggregated sequence of ISIs into temporal windows). However, this differentiation leaves room for ISI fluctuations in both types of neurons, which resemble stochastic noise.

Because of the length of our ISI sequences, we could not analyze whether there were nonlinear features.

3. In Fig. 4, why use a linear or a quadratic fitting in the scatter plots between metrics, when it is clear that a more complicated non-linear relation is observed in panels A and J for instance.

R3: We agree with the reviewer that in some cases, a more complicated nonlinear relation is observed. In order to quantify this – as suggested by the reviewer – we have changed Fig. 4 (see our reply **R3** to reviewer 1), looking for the best fits under linear and logarithmic scales. We found some linear and nonlinear relationships as can be seen in the new Fig. 4.

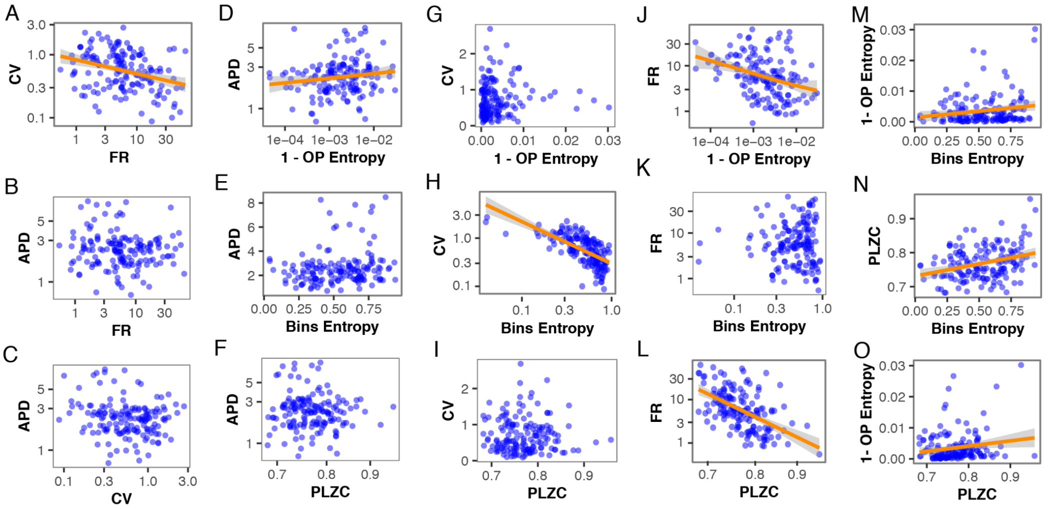

(New) **Figure 4** – The best fit for the values in panel A happens in log-log scale, meaning that a nonlinear relationship exists between CV and FR, with an exponent β_A = -0.50 (i.e., CV proportional to FR$^{\beta}$) and a Pearson correlation coefficient of r = -0.32. Similarly, the best fit for the values in panels D, H, J, and L also happens in the log-log scale, where the respective exponents are β_D = 0.46 [i.e., APD ∝ (1 - OP entropy)$^{\beta}$] with r = 0.18, β_H = -1.59 [i.e., CV ∝ (bin entropy)$^{\beta}$] with r = -0.65, β_J = -53.55 [i.e., FR ∝ (1 - OP entropy)$^{\beta}$] with r = -0.25, and β_L = -9.27 [i.e., FR ∝ (PLZC)$^{\beta}$] with r = -0.54. Contrary, the best fit for panels M, N, and O happens in a linear scale, meaning linear relationships of slopes α_M = 4.11x10-3 with r = 0.17 , α_N = 0.07 with r = 0.31, and α_O = 0.02 with r = 0.16, respectively.

4. I believe that a surrogate analysis is lacking. By comparing the present results against those obtained by a surrogate analysis could help to be more conclusive to the underlying dynamics.

R4: We have included a surrogate analysis of the OP entropy and PLZC, which shows the below values (Supp. Fig. 1). where the values for the OP entropy and PLZC in both nuclei are significantly different from their surrogates. We have modified the manuscript accordingly and placed the figure in a Supplementary Information.

Av. Gral. Flores 2125 - 11800 Montevideo Uruguay
- (598) 29243414-3234

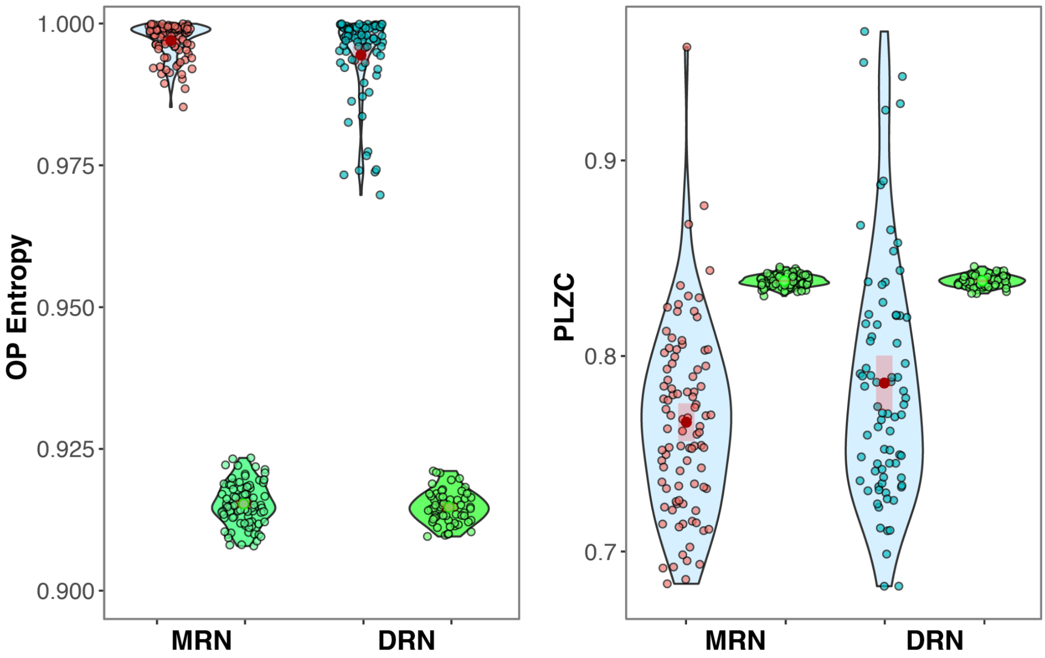

Supplementary Figure 1 – Comparison between OP Entropy (left panel) and PLZC (right panel) values from the ISI sequence of all the neurons from the MRN and DRN nuclei (from Fig. 2), and the corresponding surrogate values, which are shown in green. OP entropy and PLZC values are significantly different from those obtained by the inter-spike interval surrogates ($p < 0.001$).

Av. Gral. Flores 2125 - 11800 Montevideo Uruguay
- (598) 29243414-3234
